# Detection of Hematopoietic Stem Cell Transcriptome in Human Fetal Kidneys and Kidney Organoids Derived From Human Induced Pluripotent Stem Cells

**DOI:** 10.3389/fcell.2021.668833

**Published:** 2021-06-11

**Authors:** Jin Wook Hwang, Christophe Desterke, Julien Loisel-Duwattez, Frank Griscelli, Annelise Bennaceur-Griscelli, Ali G. Turhan

**Affiliations:** ^1^INSERM U935/UA09, Université Paris-Saclay, Villejuif, France; ^2^ESTeam Paris Sud, Université Paris Sud, Villejuif, France; ^3^INSERM U1195, AP-HP, Service de Neurologie, Faculté de Médecine, Hôpital Bicêtre, Université Paris-Saclay, Le Kremlin-Bicêtre, France; ^4^INGESTEM National IPSC Infrastructure, Villejuif, France; ^5^Division of Hematology, AP-HP Paris Saclay, Le Kremlin-Bicêtre, France

**Keywords:** hematopoietic stem cell, fetal kidney, IPSC, organoid, transcriptome

## Abstract

**Background:**

In mammalians, hematopoietic stem cells (HSCs) arise in the dorsal aorta from the hemogenic endothelium, followed by their migration to the fetal liver and to the bone marrow. In zebrafish, the kidney is the site of primary hematopoiesis. In humans, the presence of HSCs in the fetal or adult kidney has not been established.

**Methods:**

We analyzed the presence of HSC markers in the human fetal kidneys by analysis of single-cell datasets. We then analyzed in kidney organoids derived from induced pluripotent stem cells (iPSCs) the presence of hematopoietic markers using transcriptome analyses.

**Results:**

Twelve clusters were identified as stromal, endothelial, and nephron cell type-specific markers in the two fetal stage (17 weeks) kidney datasets. Among these, the expression of hematopoietic cells in cluster 9 showed an expression of primitive markers. Moreover, whole transcriptome analysis of our iPSC-derived kidney organoids revealed induction of the primitive hematopoietic transcription factor RUNX1 as found in the human fetal kidney cortex.

**Conclusion:**

These finding support the presence of cells expressing HSC transcriptome in the human kidney. The mechanisms of the appearance of the cells with the same transcriptional features during iPSC-derived kidney organoid generation require further investigation.

## Introduction

Hematopoietic stem cells (HSCs) are characterized by their capacity of both self-renewal and differentiation into blood and immune cell lineages throughout the life of the individual in a stem cell-regulating microenvironment, or HSC niche. HSC-niche interactions in the bone marrow, liver, and kidney have been extensively studied using vertebrate animal models, including mice, frogs, zebrafish, and chickens (Mikkola and Orkin, 2006; Gao et al., 2018). During mammalian hematopoiesis, the most primitive hematopoietic cells migrate from the aorta–gonad–mesonephros (AGM) region to the fetal liver and to the bone marrow, which is the site of adult hematopoiesis (Gao et al., 2018). However, the persistence of some degree of hematopoietic activity in adult tissues is possible, as this has been suggested by the discovery of donor-derived chimeric hematopoiesis after liver transplantation showing the contribution of donor-derived cells to hematopoiesis (Taniguchi et al., 1996). To our knowledge, there has been no study analyzing the possibility of donor-derived hematopoiesis after kidney transplantation. It should be reminded that in the majority of cases, kidney transplants are performed using kidneys from deceased donors (Stachura et al., 2009; Butler et al., 2018). HSC-kidney niche interactions have been studied in many reports. For example, zebrafish kidney stromal cell lines can support and maintain early hematopoietic precursors and differentiation of lymphoid, myeloid, and erythroid precursors (Stachura et al., 2009).

Recent advances in single-cell RNA sequencing technology are leading to new discoveries and validation in fetal organs and organoids (Butler et al., 2018; Menon et al., 2018; Park et al., 2018). Here, we first analyzed the presence of transcriptional markers of HSCs in fetal kidneys through analysis of a single-cell dataset described by Butler et al. (2018), Lindström et al. (2018), Menon et al. (2018), and Park et al. (2018). We then performed a transcriptome analysis of induced pluripotent stem cell (iPSC)-derived kidney organoids. We show that HSC-related markers can be detected in both fetal kidneys and the human iPSC-derived kidney organoids.

## Results

### Human Fetal Kidney Cortex Harbors Cells Expressing Hematopoietic Transcripts

**FIGURE 1:**
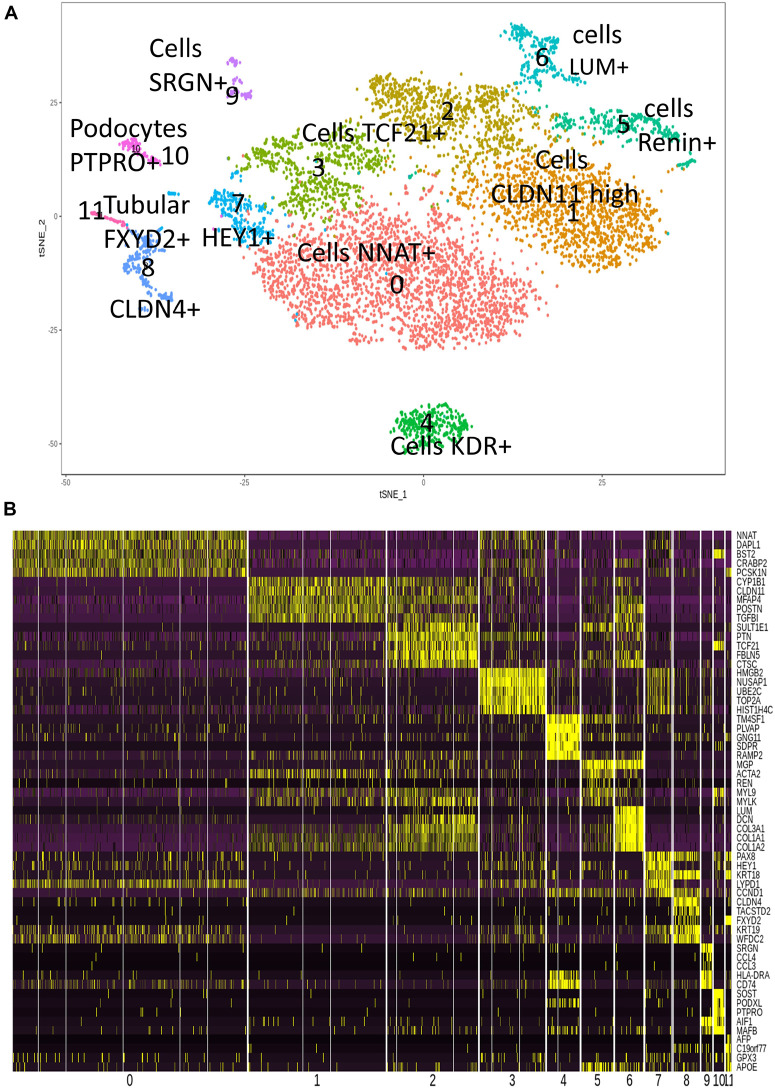
Cell heterogeneity in human fetal kidney cortex by single-cell transcriptome. **(A)** t-Distributed stochastic neighbor embedding (tSNE) plot with 11 cell clusters from the combined analysis of the merged fetal kidney cortex (two human kidneys, 17 weeks; 7,860 cells) after canonical correlation. **(B)** Heatmap with the expression pattern of the top five cluster-specific genes in 11 clusters identified in human fetal kidney cortex.

**FIGURE 2:**
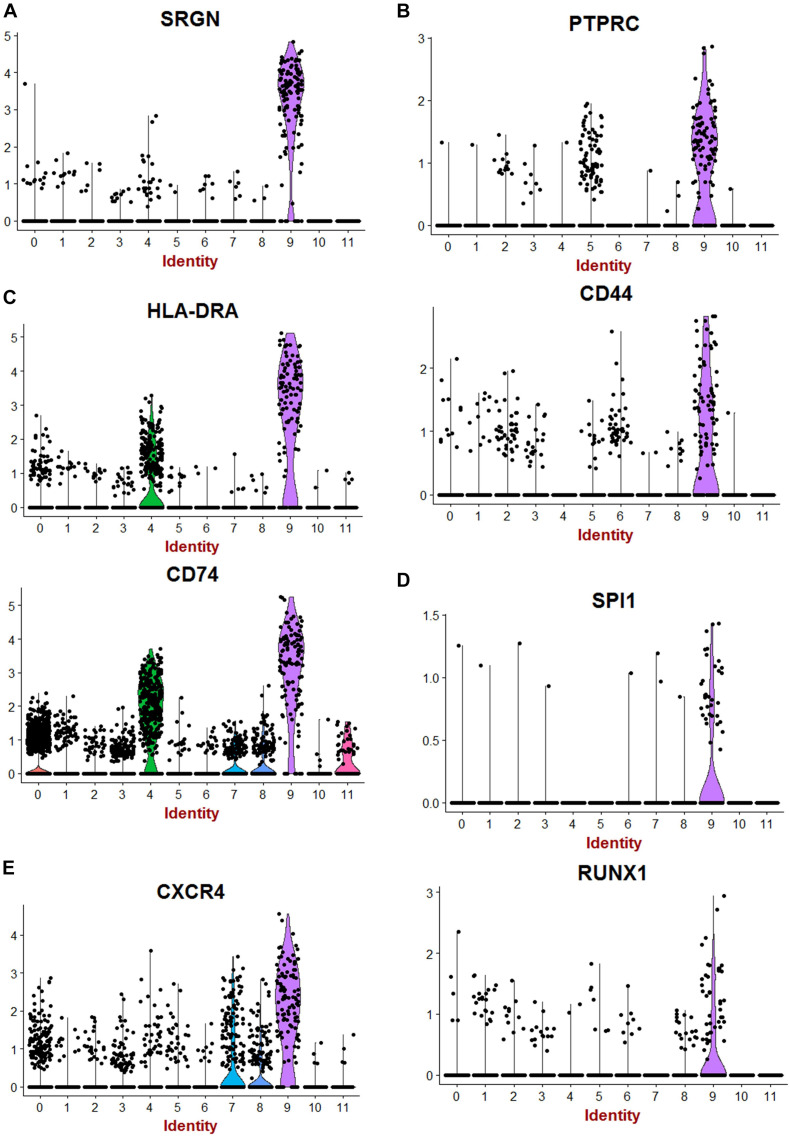
Hematopoietic transcripts detected in human fetal kidney cortex by single-cell RNA sequencing. **(A)** Violin plot of serglycin (SRGN) expression. **(B)** Violin plot of hematopoietic clusters of differentiation (PTPRC Alias CD45). **(C)** Violin plot of expression of transcripts of differentiated hematopoietic cells (HLA-DRA:HLA-DR Alpha). **(D)** Violin plot of expression of SPI1 (PU.1) and RUNX1. **(E)** Violin plot of expression for CXCR4 receptor.

Single-cell transcriptome is a powerful technology to investigate cell heterogeneity in a tissue. Butler et al. (2018), Lindström et al. (2018), Menon et al. (2018), and Park et al. (2018) performed these experiments in cortex tissues isolated from two human fetal kidneys (17 weeks) by 10× genomics technology. This work allowed us to perform *in silico* analyses. To this end, we merged and analyzed with Seurat package the 2 respective MTX files generated by cell ranger in order to suppress batch error and to perform downstream unsupervised analysis. To build the common matrix of the two samples, genes that were found expressed in a minimum of five cells by sample were conserved. After the data from the two kidney samples were merged, the Seurat digital matrix comprised 7,860 cells for 18,119 transcripts. During batch correction with canonical correlation, we observed that the two kidney samples were found well superposed in first factorial map of canonical correlation (Supplementary Figure 1A) and the shared correlation strength decreases on the 30 components of canonical correlation (Supplementary Figure 1B). t-Distributed stochastic neighbor embedding (tSNE) analysis on the common variable genes on the 40 principal components of the principal component analysis allowed to identify 12 clusters (Figure 1A) reproducible in both kidneys (Supplementary Figure 1C). Majority of the tSNE central cells comprising clusters 3, 2, 0, and 1 expressed mesoderm transcription factor TCF21 (Figure 1A and Supplementary Figure 2); also, an expression of TCF21 is positive in cells from cluster 6, which highly expressed matrix molecules such as lumican (LUM) (Figure 1A and Supplementary Figure 2), decorin (DCN), and collagens (COL3A1, COL1A1, and COL1A2) (Figure 1B). In cluster 4, cells were found to be positive for KDR (Figure 1A) and CD34 (data not shown), suggesting an expression profile corresponding to endothelial cells. Cells identified in cluster 7 have a high expression of downstream NOTCH pathway transcription factor HEY1 such as cells from cluster 0, which are central proximal from this position during tSNE analysis (Figure 1A). Some clusters of cells that are left eccentric (clusters 8 and 11) expressed tubular markers such as FXYD2 (Figure 1A and Supplementary Figure 2), encoding the sodium/potassium-transporting ATPase subunit gamma. Cluster of cells number 10, also left eccentric during tSNE analysis expressed some podocyte markers such as Protein Tyrosine Phosphatase Receptor Type O (PTPRO) (Figure 1A and Supplementary Figure 2), but also SOST (Sclerostin and PODXL: podocalyxin like) (Figure 1B). Cluster of cells number 5 expressed specifically the renin molecule a well-known renal molecule (Figure 1A and Supplementary Figure 2). These results suggest that tSNE analysis performed post canonical analysis in these two merged samples reflect the cell diversity compatible with kidney organ at this stage of development described in the original paper (Lindström et al., 2018). Surprisingly, in unsupervised tSNE analysis of the human kidney cortex, we found the left-top eccentric cluster number 9 (Figure 1A), which is principally defined by the specific expression of serglycin (SRGN) (Figures 1A, 2A and Supplementary Table 1). SRGN is known to be a hematopoietic cell granule proteoglycan. In this cluster of cells, there is also a specific expression of hematopoietic cluster of differentiation such as PTPRC alias CD45 and CD44 (Figure 2B). Some molecules such as CD74 and HLA-DRA implicated in antigen-presenting cell functionalities are also expressed in this cluster of cells (Figure 2C). Interestingly, a fraction of cells from cluster 9 also expressed primitive hematopoietic transcription factors such as SPI1 (alias PU.1) and RUNX1 (Figure 2D). Some of the cells from the same cluster also expressed CXCR4 receptor, which is well known to be expressed on primitive human hematopoietic cells for their homing function. These original results on cluster 9 suggest the presence of cells with hematopoietic transcriptome with some of them expressing primitive markers in the human kidney cortex at fetal stage (17 weeks).

### Generation and Characterization of Induced Pluripotent Stem Cell-Derived Kidney Organoids

**FIGURE 3:**
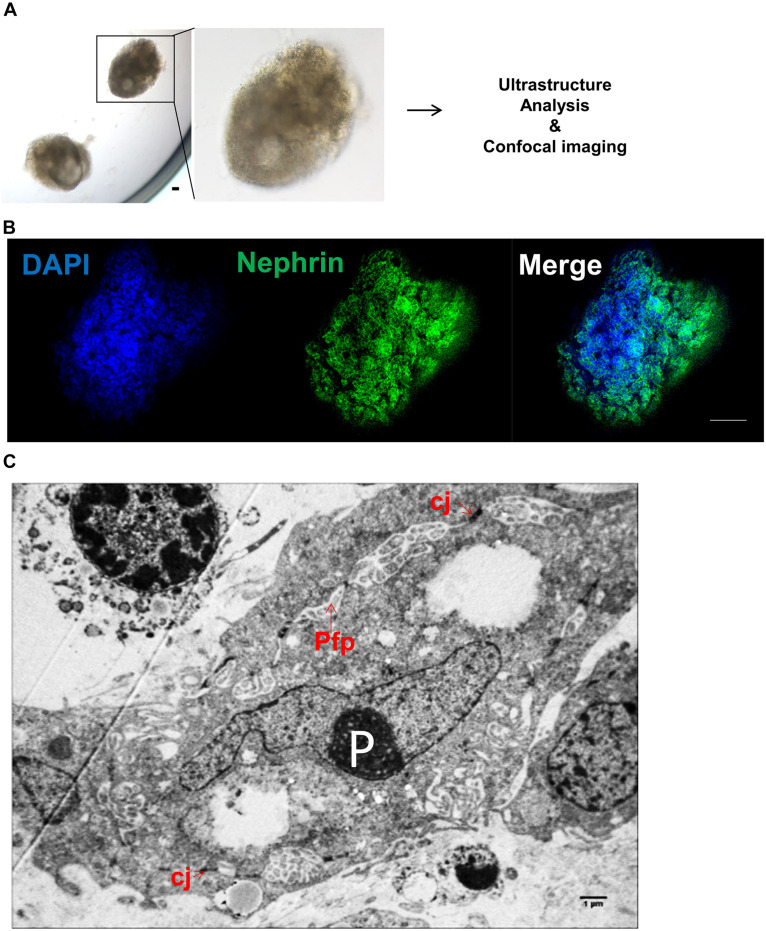
Characterization of human induced pluripotent stem cell (iPSC)-derived kidney organoids. **(A)** Optical image of iPSC-derived kidney organoids at day + 14. Scale bar: 100 μm. **(B)** Confocal analysis and whole-mount staining for Nephrin in iPSC-derived kidney organoids showing nephron vesicles. Scale bar: 50 μm. **(C)** Representative electron microscopy image of podocytes in iPSC-derived kidney organoid at day + 14 showing P, podocytes; Pfp, podocyte foot process; and cj, cell-cell junctions. Scale bar: 1 μm.

Human iPSC-derived kidney organoids have been generated as previously described (Hwang et al., 2019). Briefly, iPSC aggregates were generated in E8 media and Geltrex matrix leading to spontaneous formation of complex kidney organoids at days 12–14 of the culture (Figure 3A). We characterized iPSC-derived kidney organoids using whole-mounting staining with confocal imaging. As can be seen in Figure 3B, glomeruli-like structures, which contained cells stained for the nephron marker Nephrin, were easily identified (Ruotsalainen et al., 2000). Moreover, ultrastructure analyses revealed cell–cell junctions and the podocyte foot process formation in kidney organoids (Figure 3C).

### Detection of a Hematopoietic Transcriptome Program Induced Pluripotent Stem Cell-Derived Kidney Organoids

**FIGURE 4:**
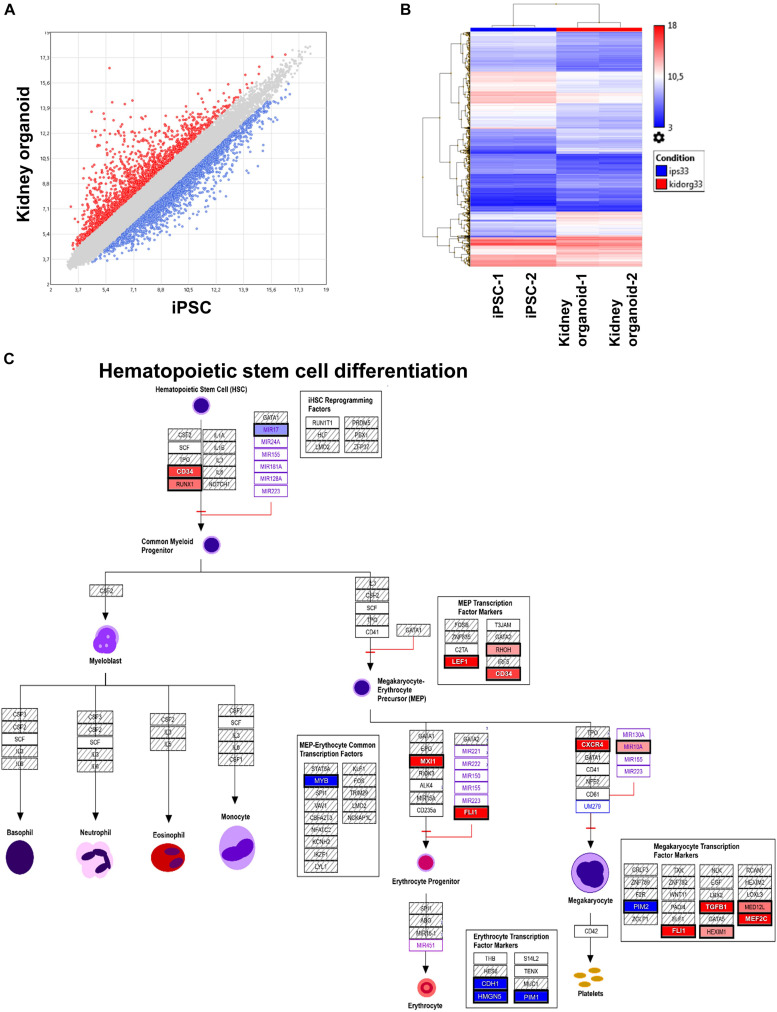
Transcriptional program induced in kidney organoid derived from human induced pluripotent stem cells (iPSCs). **(A)** Scatterplot of differentially expressed genes found in transcriptome of kidney organoid versus iPSC. **(B)** Expression heatmap with unsupervised classification performed on differentially expressed genes induced during kidney organoid differentiation from iPSC. **(C)** Functional enrichment performed on WikiPathway database showing potential implication hematopoietic stem cell function during differentiation of kidney organoid derived from human iPSC.

We performed, in duplicate, whole transcriptome analysis of iPSC-derived kidney organoids as compared with native iPSC with Clariom S human technology. After Robust Multi-array Average (RMA) normalization, we identified 3,546 differentially expressed genes (DEGs) with LIMMA algorithm (Figure 4A) comprising 1,432 upregulated genes. This DEG profile allowed to discriminate experimental sample groups by unsupervised classification (Figure 4B). After functional enrichment on WikiPathways database, we identified a hematopoietic program in iPSC-derived kidney organoids. Especially, we uncovered an upregulation of RUNX1 and CD34 corresponding to genes expressed in HSCs and that of FLI1, CXCR4, and MXI1 downstream at erythrocyte and megakaryocyte progenitor levels. There was also a repression of MYB megakaryocytic repressor (Figure 4C). These results suggest the implication of a hematopoietic transcriptional program in our iPSC-derived kidney organoids and especially induction of the primitive hematopoietic transcription factor RUNX1, which was also detectable at the single-cell level (Figure 2D) in human *ex vivo* fetal kidney cortex.

Transcriptome data of iPSCs and organoids have been submitted on National Center for Biotechnology Information (NCBI) Gene Expression Omnibus (GEO) data repository under GEO accession number Series GSE172319.

## Discussion

The involvement of kidney in hematopoiesis has been clearly demonstrated in zebrafish (Ej and Li, 2010). In humans, the most primitive hematopoietic cells arise from mesodermal lineage in AGM through hemogenic endothelium (Dzierzak and Speck, 2008). Kidney is also a tissue developed from mesoderm, but the presence of cells with HSC transcriptome has not been studied. Here, we first analyzed the HSC markers in fetal kidney through analysis of fetal kidney single-cell dataset analyses. In human fetal kidney cortex, we found some cells expressing RUNX1 in a cluster of cells that harbored expression hematopoietic markers (cluster 9 on Figures 1A,B). In cluster 9 of human fetal kidney sc-RNAseq, a high expression of hematopoietic markers was confirmed by the presence of SRGN-positive cells (Figure 2A) as well as cells expressing PTPRC alias CD45 Leukocyte Common Antigen and CD44 (receptor of hyaluronic acid) (Figure 2B). SRGN (alias hematopoietic proteoglycan core protein) is a protein found in secretory granules of myeloid cells as well as in platelets. Our analysis showed also the presence of cells positive for MHC class II molecule HLA-DRA and CD74 (Figure 2C) and, most interestingly, cells expressing of hematopoietic transcription factors SPI1 and RUNX1 (Figure 2D). Finally, in this cluster 9 of fetal kidney, we found a higher expression of CXCR4 receptor of CXCL12 implicated in migration properties of HSCs (Figure 2E). All these results allowed to suggest the presence of cells harboring hematopoietic transcriptome in human fetal kidney.

We then analyzed the transcriptome of iPSC-derived kidney organoids and performed differential expression analysis of the kidney organoid versus parental iPSCs. Microarray analysis revealed important regulation of transcriptional program of these cells during their differentiation (Figure 4A). This differentially expressed program allowed to discriminate group samples by unsupervised classification (Figure 4B). After functional enrichment performed on upregulated genes during the differentiation process of the iPSC-derived kidney organoids, we observed the induction of HSC markers such as RUNX1 and CD34 (Figure 4C). It is well established that RUNX1 along with a *cis-*regulatory elements integrating the GATA, ETS, and SCL transcriptional networks plays a major role in HSC generation (Nottingham et al., 2007). We also found induction of FLI1 during the differentiation of iPSC-derived kidney organoid. SPI1 (alias PU.1), the main target downstream RUNX1 (Imperato et al., 2015), is also a master regulator of hematopoiesis, as it prevents excessive HSC division and exhaustion by controlling the transcription of multiple cell-cycle regulators (Staber et al., 2013). Association of SPI1 and RUNX1 is comprised a combination of seven transcription factors, which are sufficient to convert hemogenic endothelium into hematopoietic stem and progenitor cells that engraft myeloid, B, and T cells in primary and secondary mouse recipients (Sugimura et al., 2017). In transcriptome analyses of iPSC-derived kidney organoids, we found an upregulation of MYB, which is known to participate to cell fate decisions between erythropoiesis and megakaryopoiesis in human hematopoiesis (Bianchi et al., 2015). Amongst the hematopoietic transcripts identified in human fetal kidney cortex, we have also detected the expression of which, CXCR4 in relation with its ligand CXCL12, is involved in homing of hematopoietic cells to the bone marrow (Sugiyama et al., 2006).

Our data have some limitations including the fact that we cannot exclude the presence of mesodermal cells undergoing the fate of hematopoietic differentiation during our kidney organoid differentiation. Secondly, we could not identify the presence of cells with HSC functionality (self-renewal; differentiation) in the current experiments. However, these data suggest that at some point during embryonic development, a special “kidney niche” could appear transiently in humans. The identification of such a niche or its molecular counterparts could be of major interest to amplify human HSCs for transplantation purposes, such has been described in zebrafish (Stachura et al., 2009; Wolf et al., 2017). It is known that zebrafish embryonic stromal trunk (ZEST) cells derived from the HSC emergence site are functionally similar to the mammalian AGM niche cells. Moreover, ZEST cells and kidney cell lines have similar signaling properties (Wolf et al., 2017; Mahony and Bertrand, 2019). Our results suggest that a “kidney microenvironmental niche” niche could be of interest to generate conditions for HSC culture and expansion.

## Materials and Methods

• Key resources table

**Table:** 

Reagent or resource	Source	Identifier
Antibodies		
Nephrin	Abcam	ab85379
DAPI	Sigma-Aldrich	D9542
**Chemicals, peptides, and recombinant proteins**		
Essential 8 basal medium	Thermo Fisher Scientific	A1516901
Essential 8 supplement	Thermo Fisher Scientific	A1517101
Geltrex LDEV-Free Reduced Growth Factor Basement Membrane Matrix	Thermo Fisher Scientific	A1413202
ROCK inhibitor	Global stem	GSR-6102

**Reagent or resource**	**Source**	**Identifier**

Experimental models: Cell lines		
Human iPSC: PB33	Human	–
Osmium tetroxide solution	Sigma-Aldrich	75632
Glutaraldehyde grade I	Sigma-Aldrich	G5882
**Software and algorithms**		
ImageJ		

### Generation of Induced Pluripotent Stem Cells

The iPSC line used is this study was generated using Sendaï virus-mediated gene transfer of the four “Yamanaka” factors as previously described, using bone marrow mononuclear cells of a normal donor (Hwang et al., 2019).

### Generation of Kidney Organoids

Induced pluripotent stem cells were maintained on Geltrex-coated (STEMCELL Technologies Inc., Vancouver, BC, Canada) flat culture dish in E8 media (STEMCELL Technologies Inc.) according to manufacturer’s guidelines. Colonies were manually harvested at 60–80% confluence. Cells were then collected and dissociated into single cells using EDTA. Cells (1 × 10^6^ or 1 × 10^5^/well) were put onto ultra-low attachment 24-well or 96-well plate (Corning Inc., Corning, NY, United States) to allow them to form aggregated in suspension with ROCK inhibitor (2–5 μmol). Cell aggregates were cultured in E8 medium (STEMCELL Technologies) with daily medium change for 6–7 days. Control iPSC-A (iPSC-aggregates) were plated on a Geltrex (STEMCELL Technologies) in 96-well plate or 8-well culture chamber. And then aggregates were treated E8 medium (STEMCELL Technologies) with daily medium change for 12–14 days. Images were taken using a NIKON microscope (Nikon Instruments Inc., Melville, NY, United States).

### Whole-Mount Immunostaining of 3D Kidney Organoids

Kidney organoids cultured on 96-well culture dishes were washed with phosphate-buffered saline (PBS), fixed with 4% paraformaldehyde in PBS for 120 min, permeabilized with 0.2% Triton X-100 (Sigma) in PBS, and blocked with 10% serum. For Nephrin staining, the antibody [Nephrin (Cat#ab85379; Abcam), Cambridge, United Kingdom] was diluted in PBS containing 10% serum and washed in PBS. Samples were incubated with secondary antibodies in antibody dilution buffer and then washed in PBS. Nuclei were labeled with DAPI mounting medium. Visualization and capture were realized with a Zeiss confocal microscope. Negative controls were the experiments with secondary antibodies, yielding no staining (data not shown).

### Transmission Electron Microscopy

Kidney organoids were gently centrifuged and pelleted before the TEM process, as follows. The cells were fixed in 2.5% glutaraldehyde in PBS for 1 h at 4°C, washed in PBS, and fixed in 1% osmium tetroxide in PBS for 1 h. They were dehydrated in ascending series of graded ethyl alcohols and then in acetone. Each sample was infiltrated with the resin before being embedded in epoxy resin and polymerized for 72 h. Semi-thin sections of about 0.5–1 μm were obtained and colored with toluidine blue before being examined via a light microscope with an associated digital camera and hooked to a computer for image processing and editing (Leica DC300, Wetzlar, Germany). Ultra-thin sections of about 60/90 nm were contrasted with heavy metals (uranyl acetate and lead citrate) and were examined using a Jeol 1010 transmission electron microscope at an accelerated voltage of 80 kV. Images were photographed on digital images Gatan Digital Micrograph (brure Erlangen 500 W) and camera and edited by ImageJ and Microsoft Power Point.

### Human Fetal Single-Cell Transcriptome Analysis

Dataset GSE112570 of single-cell RNA-sequencing allows to explore cellular heterogeneity of human kidney cortical nephrogenic niche (Lindström et al., 2018). Experiments were performed with technology 10× Genomics single-cell RNA sequencing on two human kidney samples (17 weeks) indexed in GEO database: GSM3073088 and GSM3073089. Molecular index was realized: Chromium Single Cell 3′ v2 single-cell RNA-Seq of poly A selected mRNA kit (10× Genomics), and sequencing was processed on NextSeq 500 (Illumina, San Diego, CA, United States). Bioinformatics base call by bcl2fastq v. 2.17 reads were mapped using STAR 2.5.1b (Genome: GRCh37), and count tables were generated using the Cell Ranger software version 1.3.1. Downstream bioinformatics single-cell transcriptome analyses were performed in R software version 3.4.3. Digital matrix was built with both 10× MTX files and merged in Seurat R-package version 2.3.0 (Butler et al., 2018) with package dependencies of matrix version 1.2-12, cowplot 0.9.2, and ggplot2 version 2.2.1 (Wickham, 2009). Batch correction was performed with canonical correlation on 30 dimensions before mathematical dimension reduction with tSNE algorithm. Also, dplyr library version 0.7.4 was used to generate intermediate table of best genes by cluster. Bioinformatics code to perform these single-cell analyses was deposed at the following web address: https://github.com/cdesterke/hsckidney/.

### Kidney Organoid Microarray Analysis

Microarray Clariom S human was done on process total RNA from human wild-type (WT) iPSC and its derived kidney organoids in duplicates (Hwang et al., 2019). Expression matrix was built with CEL files generated on Affymetrix Station and normalized by RMA method with TAC version 4.0 software (Applied Biosystems, Foster City, CA, United States) (Irizarry et al., 2003). DEGs were estimated with linear models for microarray data (LIMMA) algorithm by using a false discovery rate threshold of less 5% (Ritchie et al., 2015). Functional enrichment analysis on DEGs was performed on WikiPathway database.

## Data Availability Statement

The data presented in the study are deposited in the Gene Expression Omnibus (GEO) repository, accession number GSE172319.

## Author Contributions

JH and AT conceived, designed, analyzed the data, and wrote the manuscript. JH performed all organoids experiments and performed confocal laser scanning microscopy with analysis. JL-D performed the TEM. CD analyzed the bioinformatics data. AB-G, FG, and AT analyzed the data and supervised the project. JH, CD, and AT wrote the manuscript. All authors contributed to the article and approved the submitted version.

## Conflict of Interest

The authors declare that the research was conducted in the absence of any commercial or financial relationships that could be construed as a potential conflict of interest.

## Supplementary Material

Supplementary Figure 1 Canonical correlation with batch correction between the two fetal kidneys process in single cell transcriptome.**(A)** Factorial map showing superposition of the cells from the respective kidneys. During normalization of the transcriptome, a canonical correlation normalization was applied between the cells of the 2 kidneys, the factorial map showed that cells from each kidney were well spread on this map with a good overlapping. **(B)** Profile of the shared correlation strength between the respective kidneys (30 dimensions); tSNE plot post canonical correlation on the merged 2 kidneys. **(C)** t-SNE dimension reduction of single cell transcriptome from human fetal kidney after batch correction, cells from each kidney (respectively green and red) are plotted with t-SNE dimension reduction algorithm and their distribution in the map confirmed a good batch correction during the analyses (canonical correlation with Seurat R-package).

Supplementary Figure 2 Violinplot of cluster markers found in single cell sequencing after merging the fetal cortex of two human kidneys.

Supplementary Table 1 Table presenting differential expressed genes found in cluster 9 as compared to others clusters in human fetal kidney cortex.
