# Supplementary figures and images for: Detection of Hematopoietic Stem Cell Transcriptome in Human Fetal Kidneys and Kidney Organoids Derived From Human Induced Pluripotent Stem Cells
Source: Front Cell Dev Biol. 2021 Jun 11;9:668833. doi: 10.3389/fcell.2021.668833 (PMC8226023; doi:10.3389/fcell.2021.668833)

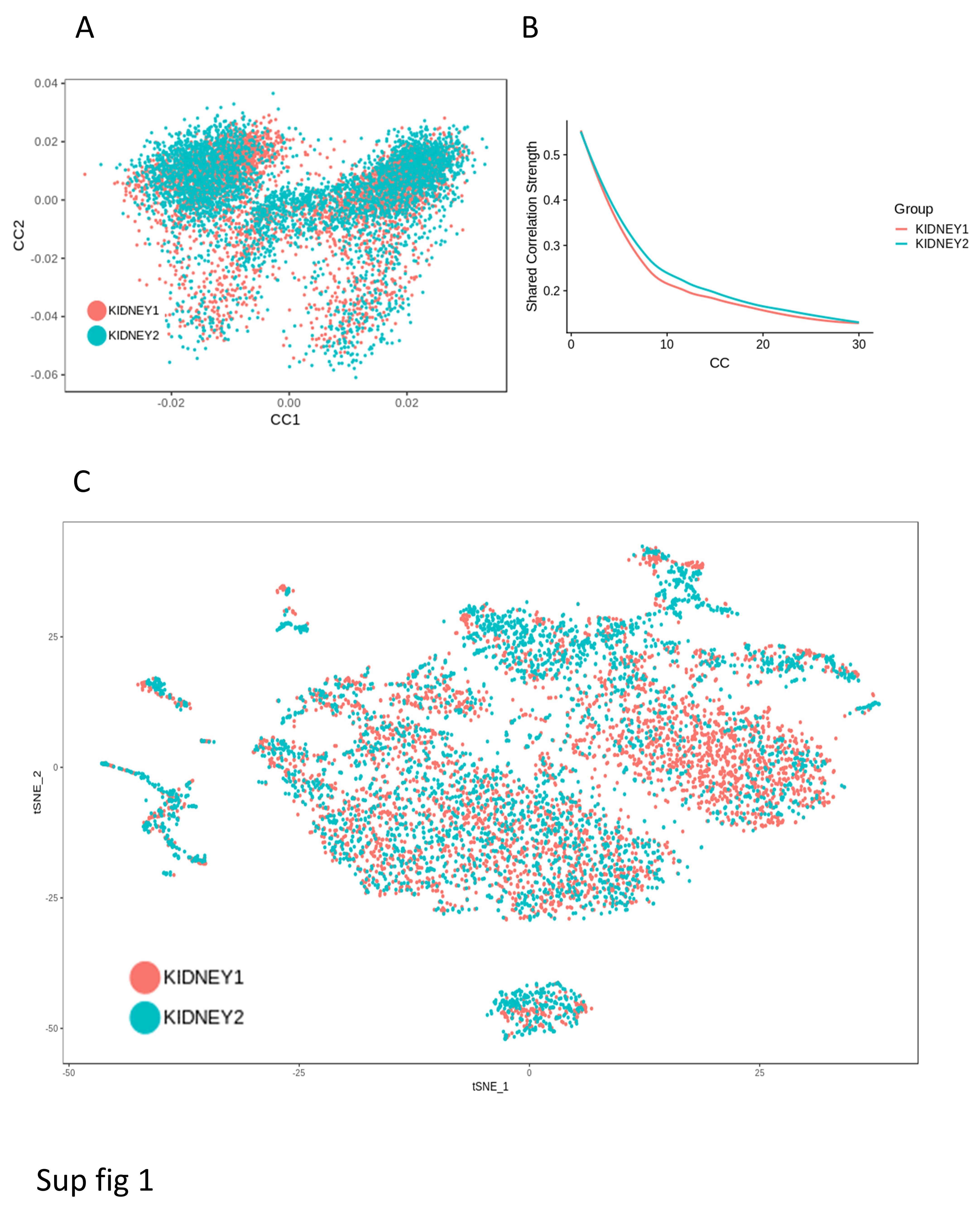

Supplement: Supplementary Figure 1 — Canonical correlation with batch correction between the two fetal kidneys process in single cell transcriptome.(A) Factorial map showing superposition of the cells from the respective kidneys. During normalization of the transcriptome, a canonical correlation normalization was applied between the cells of the 2 kidneys, the factorial map showed that cells from each kidney were well spread on this map with a good overlapping. (B) Profile of the shared correlation strength between the respective kidneys (30 dimensions); tSNE plot post canonical correlation on the merged 2 kidneys. (C) t-SNE dimension reduction of single cell transcriptome from human fetal kidney after batch correction, cells from each kidney (respectively green and red) are plotted with t-SNE dimension reduction algorithm and their distribution in the map confirmed a good batch correction during the analyses (canonical correlation with Seurat R-package). [file Image_1.JPEG]

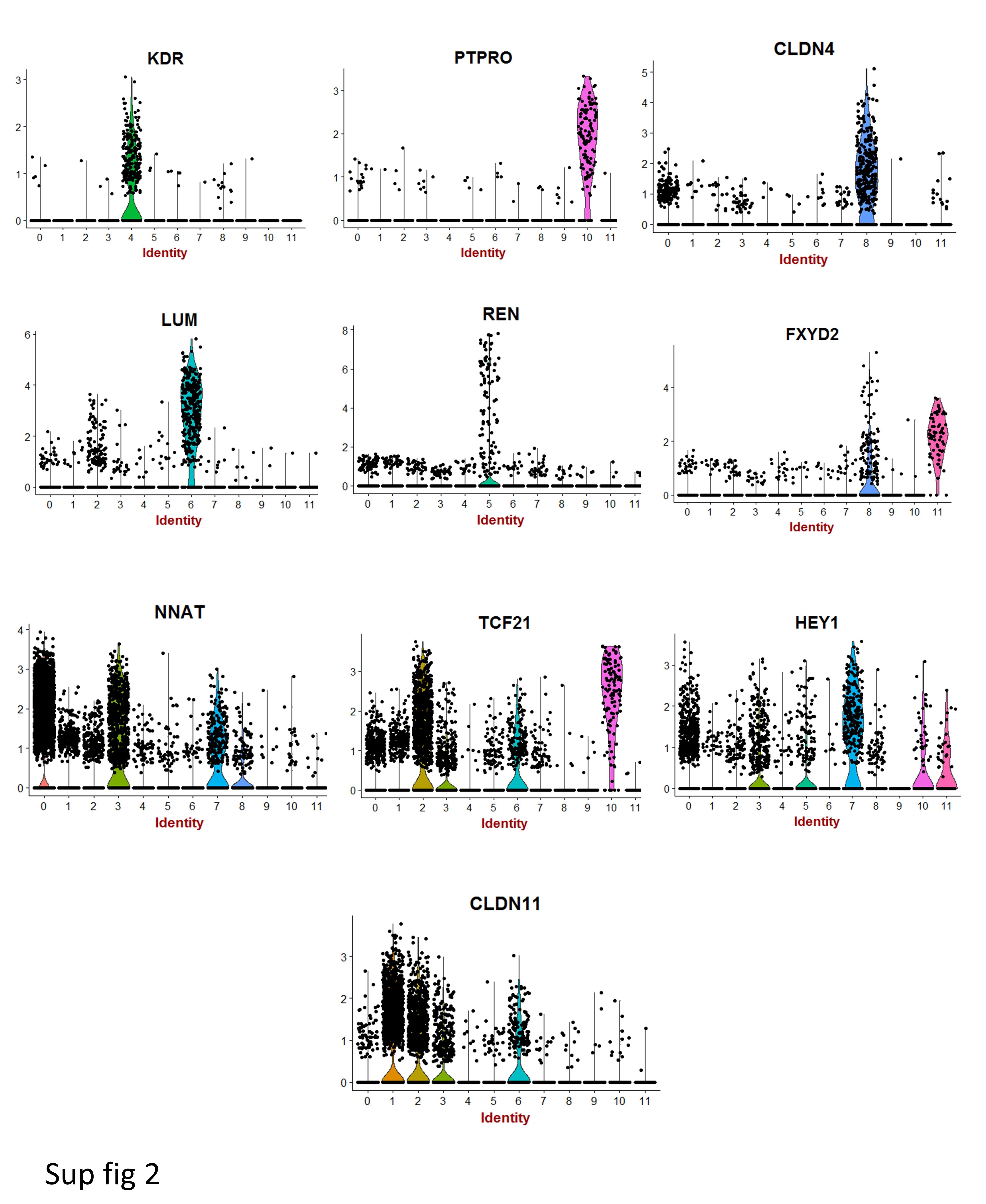

Supplement: Supplementary Figure 2 — Violinplot of cluster markers found in single cell sequencing after merging the fetal cortex of two human kidneys. [file Image_2.JPEG]
